# The U-shaped incision and diagonal suturing of the sellar dura in endoscopic transsphenoidal pituitary adenoma resection

**DOI:** 10.1007/s00701-026-06889-1

**Published:** 2026-04-28

**Authors:** Yikun Xie, Bin Han, Gengshi Gu, Haitao Wang

**Affiliations:** 1 Jining Medical University, Jining, China; 2 Department of Neurosurgery, The Affiliated Hospital of Jining Medical University, Jining, 272029 Shandong China

**Keywords:** Endoscopy, Cerebrospinal fluid leakage, Sellar dura mater diagonal suture, Pituitary adenoma

## Abstract

**Background:**

Endoscopic endonasal transsphenoidal (EET) approach has been widely utilized for the treatment of pituitary adenoma. However, postoperative cerebrospinal fluid (CSF) leakage is still a concern. One of the key surgical points to prevent postoperative CSF leakage is how to reduce the defect of the sellar floor and increase its support.

**Method:**

We presented a step-by-step description of the incision method of the sellar dura during endoscopic transsphenoidal pituitary adenoma resection, as well as the sellar dura mater diagonal suture technique to reduce postoperative sellar dura mater defects and increase the support of the sellar floor. These methods have been widely applied in our surgical procedures.

**Conclusion:**

These measures effectively increase the support of the sellar floor and reduce the risk of postoperative CSF leakage in cases of low cerebrospinal fluid leakage during surgery, and are easy to perform.

**Supplementary Information:**

The online version contains supplementary material available at 10.1007/s00701-026-06889-1.

## Relevant surgical anatomy

EET approach is a commonly performed neurosurgical technique that has been widely utilized for the treatment of pituitary adenoma [9]. Cerebrospinal fluid (CSF) leakage after endoscopic endonasal surgery (EES) is a common complication, with an incidence ranging from 1.6% to 40% [5, 6, 8]. CSF leakage can lead to serious postoperative complications such as intracranial infection and pneumocephalus. The degree of intraoperative CSF leak detected is classified into 4 levels: grade 0, grade 1, grade 2 and grade 3 [1]. To avoid postoperative CSF leakage, various measures should be taken based on the different grades of intraoperative CSF leakage, such as multi-layer reconstruction with fat packing, fascia lata, artificial dura mater, and pedicled nasal septal flap (NSF) [3]. The purpose of these measures is to reduce and ultimately close the cranial base defect, while providing sufficient support to promote healing and prevent postoperative CSF leakage. Among them, grade 1 leakage refers to small or “weeping” CSF flow without a visible diaphragm defect and is recommended to be repaired with synthetic allograft underlays and overlays with an intrasellar pack of autologous abdominal fat [4, 10].

**Fig. 1:**
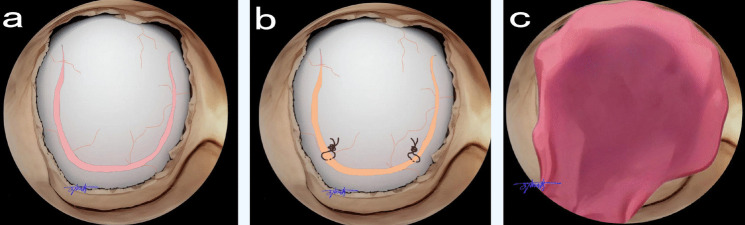
Illustration of diagonally suturing for U-shaped opening of the sellar dura. **a** U-shaped incision over the sellar dura. **b** U-shaped dural flap was turned back inferiorly and secured to lower dural edge. **c** Pedicled NSF rotated superiorly and coverd the entire defect

We routinely use a U-shaped incision to open the sellar floor dura before pituitary tumor resection (Fig. 1a). If there is Grade 1 intraoperative CSF leaks after tumor removal, diagonal sutures are performed on the sellar floor dura (Fig. 1b), followed by the application of a vascularized nasoseptal flap (NSF) (Fig. 1c), without using fat packing or fascia lata, resulting in a postoperative CSF leak rate of less than 0.5%.

## Description of the technique (Video 1)

### Patient positioning

After undergoing general anesthesia, the patients were positioned in supine position with their head fixed in a head holder. The upper body was elevated by 20—30°, while the head was slightly extended and rotated to the right by 5°.

### Nasal and sphenoidal steps

After nasal disinfection, a 0° endoscope was placed in the right nostril. Vascularized NSF is harvested from the right nasal cavity and repositioned towards the nasopharynx. Eggshell technique was used to remove the bone covering the sella turcica.

### Dural opening

**Fig. 2:**
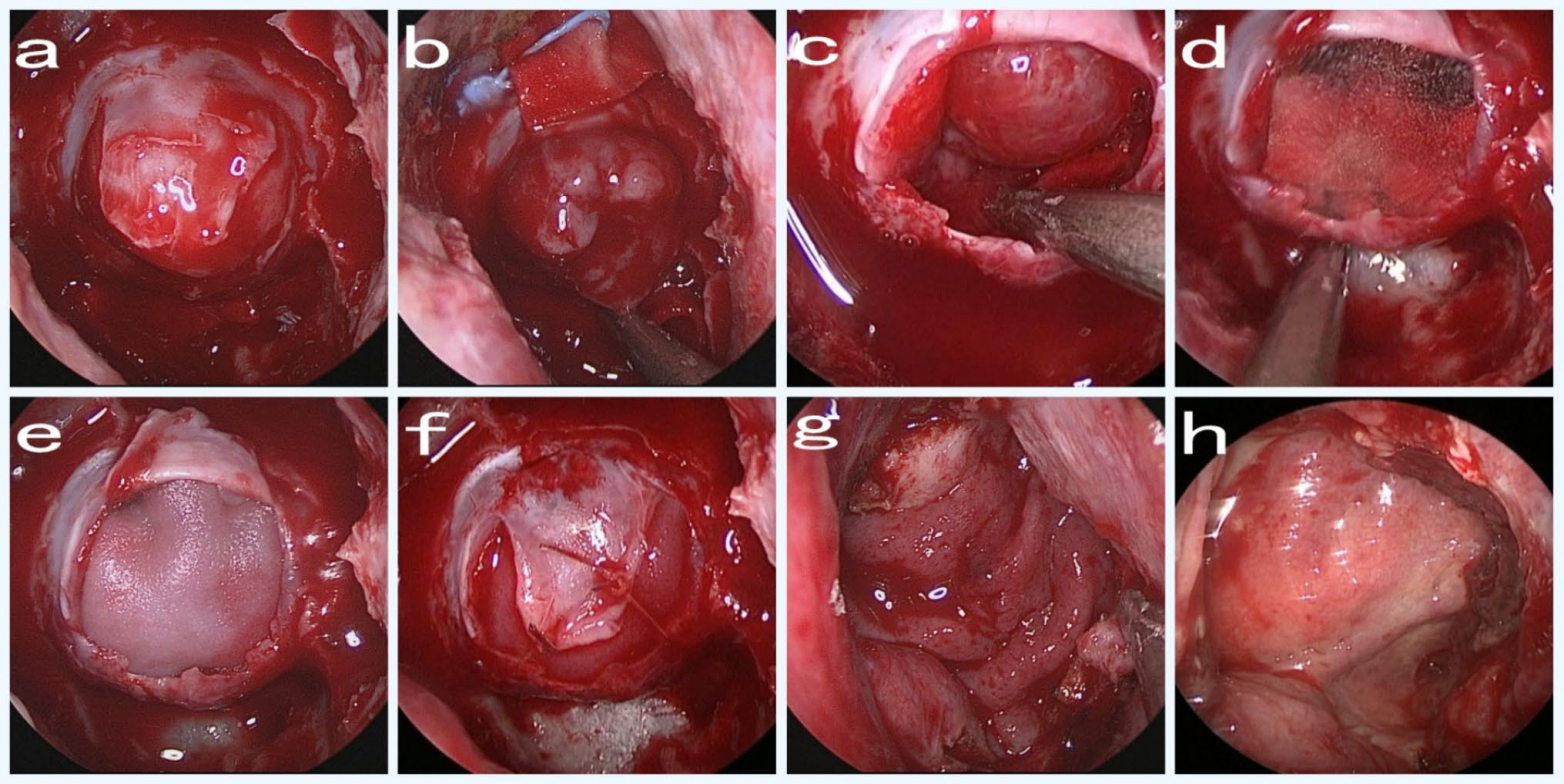
Procedures for skull base reconstruction. **a** U-shaped incision over the sellar dura. **b** U-shaped dural flap was turned over to the tuberculum sellae and temporarily fixed to the bone with neuro patties. **c** After tumor resection, grade 1 CSF leakage was observed. **d** Hemostatic fibers and gelatin sponge filled the sella turcica. **e** Place the artificial dura on the inner side of the sellar dura defect. **f** U-shaped dural flap was turned back inferiorly, and the two corners of dural flap were sutured to the posterior edge of dural defect. **g** Pedicled NSF coverd the entire defect. **h** The NSF healed well without CSF leakage after the removal of the iodine gauze 2 weeks after operation

A U-shaped incision over the sellar dura was made near the inner edge of the cavernous sinuson both sides to form the dural flap (Fig. 2a), which was then turned over to the tuberculum sellae and temporarily fixed to the bone with neuro patties (Fig. 2b).

### Skull base reconstruction with dural suturing

After tumor resection, grade 1 CSF leakage with “weeping” CSF flow was observed (Fig. 2c). The first step in repairing a CSF leak was to fill the saddle area with hemostatic fibers and gelatin sponge to eliminate the dead space (Fig. 2d). In the second step, an artificial dura mater with a diameter larger than the sellar dura defect was placed into the medial side of the edge of the dura defect, which obliterated the potential space in dura defect while creating a laminar surface for further reconstruction (Fig. 2e).

Next, the U-shaped dural flap was turned back inferiorly, and the two corners of its distal end were sutured to the posterior edge of dural defect using the “sliding-lock-knot” technique that the knot was made outside the nasal cavity and then slid into the operative region (Fig. 2f). The purpose of diagonally suturing for U-shaped opening of the sellar dura was to reduce the defect area of the sellar floor dura and to provide adequate anti-pressure and counter-shift capability, although this is not an water-tight closure. Subsequently, NSF was rotated superiorly to cover the dural closure and bony skull base defect (Fig. 2g). Finally, the nasal cavity was filled with iodine gauze.

### Prognostic evaluation

Continuous monitoring was necessary post-surgery to determine the presence of CSF rhinorrhea. After 2 weeks, the endoscopic removal of the iodine gauze took place [7], allowing for the evaluation of both the healing of the NSF and the CSF leakage (Fig. 2h).

## Indications

This method is well-suited for transnasal endoscopic pituitary adenomas resection, and its role in preventing postoperative cerebrospinal fluid leakage is more suitable for cases where grade 1 cerebrospinal fluid leakage occurs during surgery, without the need for additional use of fat packing or fascia lata.

## Limitations

For grade 2 intraoperative CSF leaks, we have also attempted to use this method with equally satisfactory results. However, given the presence of obvious sellar diaphragm defects in grade 2 and higher grade intraoperative CSF leaks, if the NSF does not heal well, there is still a risk of postoperative CSF leaks. Therefore, it is currently not recommended for cases of grade 2 and above intraoperative CSF leaks unless it can be confirmed that the blood supply to the NSF is relatively sufficient and ensures a smooth healing process.

## How to avoid complications

Considering the distribution of the sphenopalatine artery and its branches within the mucosa at the incision site, electrocoagulation is advisable during mucosal incision to reduce the risk of delayed nasal hemorrhage.

Avoid excessive filling of gelatin sponge to prevent postoperative visual impairment caused by high intrasellar pressure.

In cases of intraoperative CSF leakage, it was necessary to administer intravenous ceftriaxone (4 g, once daily, recommandation level 1B) [2] for at least 48 h postoperatively to prevent intracranial infection.

## Specifc information for patient

Patients must be informed about the risks of postoperative common complications after endoscopic endonasal surgery.

## Key points

1. The edges on both sides of the saddle bottom bone window should reach the inner edge of the cavernous sinus.
2. The upper edge of the saddle base bone window should reach the level of the tuberculum sellae as much as possible.
3. When performing the U-shaped incision of the sellar floor dura, it is advisable to keep the two sides as close as possible to the inner edge of the cavernous sinus, while being careful not to damage the cavernous sinus and the internal carotid artery.
4. Based on the preoperative imaging assessment, caution must be exercised during the incision of the U-shaped dura to avoid harm to the superior diaphragmatic reflection of the saddle, thereby preventing significant CSF leakage.
5. The U-shaped sellar floor dura was elevated towards the tuberculum sellae and secured in place with neuro patties. This procedure is important in in safeguarding the surgical field from interference during tumor removal.
6. After tumor resection, a hard artificial dura mater could be used to provide better support than a soft artificial dura mater in the medial side of the sellar dura defect. If the visible leakage of CSF becomes more pronounced, a collagen matrix may also be positioned within the subdiaphragmatic space as an inlay graft.
7. The excessive use of hemostatic fibers and gelatin sponge for packing within the saddle region should be avoided to prevent an increase in intrasaddle pressure.
8. Suture of the sellar dura mater should be performed with attention to the diagonal direction, tightening the suture line to reduce the sellar dura defect. However, care should be taken to avoid excessive suture tension to prevent exacerbation of postoperative headaches.
9. Mastering the "sliding-lock-knot" technique proficiently is essential for ensuring the smooth completion of dural suturing process and its efficacy.
10. Successfully preparing NSF is crucial for ensuring its blood supply and smooth healing process.

## Supplementary Information

Below is the link to the electronic supplementary material.

Supplementary file1 (MP4 145631 KB)

## Data Availability

No datasets were generated or analysed during the current study.

## Code availability

The code used in this article is available from the corresponding author on reasonable request.

## Declarations

### Disclaimer

The funders had no role in any aspect of the perioperative procedures; in the writing of the manuscript, or in the decision to publish the results.
